# Supplementary material for: Transcriptomic Insights into Mechanisms of Early Seed Maturation in the Garden Pea (Pisum sativum L.)
Source: Cells. 2020 Mar 23;9(3):779. doi: 10.3390/cells9030779 (PMC7140803; doi:10.3390/cells9030779)

Zhongwan−6,25(2)

Global Summary

%DE = 0.28

genes with fdr < 0.2 = 166 (71 + / 95 −)

genes with fdr < 0.1 = 157 (66 + / 91 −)

genes with fdr < 0.05 = 150 (64 + / 86 −)

genes with fdr < 0.01 = 129 (54 + / 75 −)

genes in genesets = 0

<FC> = 0

<t−score> = 0

<p−value> = 0

<fdr> = 0.72

Global Genelist

Rank	ID	log(FC)	p−value	fdr	Metagene	Description
1	TRINITY_DN2	−0.17	2e−16	2e−15	14 x 3	
2	TRINITY_DN1	5.71	2e−16	2e−15	20 x 1	
3	TRINITY_DN1	3.01	2e−16	2e−15	20 x 20	
4	TRINITY_DN5	0.7	2e−16	2e−15	2 x 20	
5	TRINITY_DN5	0.27	2e−16	2e−15	13 x 14	
6	TRINITY_DN1	−0.12	2e−16	2e−15	10 x 1	
7	TRINITY_DN5	0.12	2e−16	2e−15	1 x 11	
8	TRINITY_DN2	2.73	2e−16	2e−15	20 x 1	
9	TRINITY_DN2	1.3	2e−16	2e−15	20 x 1	
10	TRINITY_DN3	−0.27	2e−16	2e−15	1 x 1	
11	TRINITY_DN3	0.16	2e−16	2e−15	20 x 17	
12	TRINITY_DN5	0.2	2e−16	2e−15	1 x 1	
13	TRINITY_DN5	−0.15	2e−16	2e−15	14 x 4	
14	TRINITY_DN4	−0.45	2e−16	2e−15	15 x 3	
15	TRINITY_DN4	−1.14	2e−16	2e−15	1 x 1	
16	TRINITY_DN4	1.68	2e−16	2e−15	18 x 20	
17	TRINITY_DN1	−0.17	2e−16	2e−15	1 x 1	
18	TRINITY_DN2	−0.17	2e−16	2e−15	1 x 2	
19	TRINITY_DN2	−0.3	2e−16	2e−15	1 x 1	
20	TRINITY_DN4	−0.52	2e−16	2e−15	1 x 1	

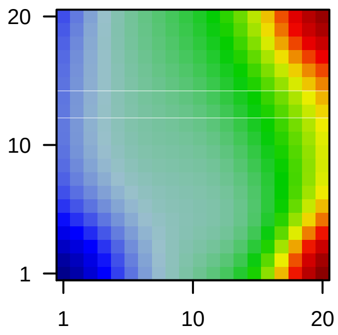

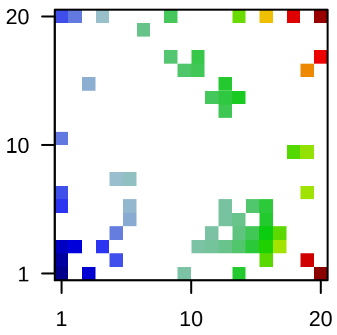

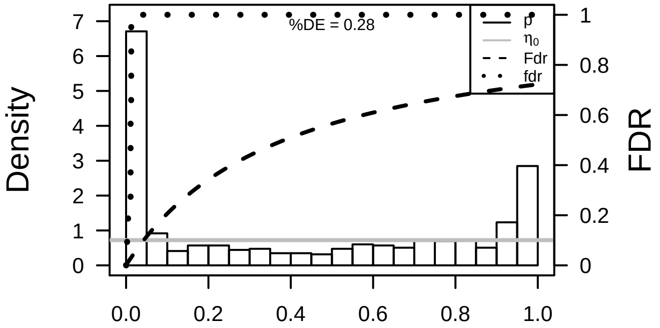

Supplement: Supplementary file 1 [file cells-09-00779-s001.zip › Supplementary materials/FileS3.zip!/FileS2/Hormones - Results/Summary Sheets - Samples/Zhongwan.6.25.2..pdf]
